# First Identification of Rare Exonic and Deep Intronic Splice-Altering Variants in Patients With Beta-Sarcoglycanopathy

**DOI:** 10.3389/fped.2022.900280

**Published:** 2022-06-22

**Authors:** Zhiying Xie, Chengyue Sun, Chang Liu, Xujun Chu, Qiang Gang, Meng Yu, Yiming Zheng, Lingchao Meng, Fan Li, Dongliang Xia, Li Wang, Ying Li, Jianwen Deng, He Lv, Zhaoxia Wang, Wei Zhang, Yun Yuan

**Affiliations:** ^1^Department of Neurology, Peking University First Hospital, Beijing, China; ^2^Department of Neurology, Peking University People’s Hospital, Beijing, China; ^3^Science and Technology, Running Gene Inc., Beijing, China; ^4^Beijing Anzhen Hospital, Capital Medical University, Beijing, China

**Keywords:** *SGCB*, aberrant splicing, intron, splice-altering variants, diagnosis

## Abstract

**Background:**

The precise genetic diagnosis of a sarcoglycanopathy or dystrophinopathy is sometimes extremely challenging, as pathogenic non-coding variants and/or complex structural variants do exist in *DMD* or sarcoglycan genes. This study aimed to determine the genetic diagnosis of three patients from two unrelated families with a suspected sarcoglycanopathy or dystrophinopathy based on their clinical, radiological, and pathological features, for whom routine genomic detection approaches failed to yield a definite genetic diagnosis.

**Methods:**

Muscle-derived reverse transcription-polymerase chain reaction analysis and/or TA cloning of *DMD*, *SGCA*, *SGCB*, *SGCD*, and *SGCG* mRNA were performed to identify aberrant transcripts. Genomic Sanger sequencing around the aberrant transcripts was performed to detect possible splice-altering variants. Bioinformatic and segregation studies of the detected genomic variants were performed in both families.

**Results:**

In patients F1-II1 and F1-II2, we identified two novel pathogenic compound heterozygous variants in *SGCB*. One is a deep intronic splice-altering variant (DISV), c.243 + 1558C > T in intron 2 causing the activation of an 87-base pair (bp) pseudoexon, and the other one is a non-canonical splicing site variant, c.243 + 6T > A leading to the partial intron inclusion of 10-bp sequence. A novel DISV, c.243 + 1576C > G causing a 106-bp pseudoexon activation, and a nonsense variant in *SGCB* were identified in compound heterozygous state in patient F2-II1. Unexpectedly, the predicted nonsense variant, c.334C > T in exon 3, created a new donor splice site in exon 3 that was stronger than the natural one, resulting in a 97-bp deletion of exon 3 (r.333_429del).

**Conclusion:**

This is the first identification of rare exonic and DISVs in the *SGCB* gene.

## Introduction

Sarcoglycanopathies consisting of four subtypes of autosomal recessive limb-girdle muscular dystrophies (LGMD2D, LGMD2E, LGMD2F, and LGMD2C) are caused by biallelic loss-of-function variants in four sarcoglycan genes, including the *SGCA*, *SGCB*, *SGCD*, and *SGCG* genes encoding the α-, β-, δ-, and γ-sarcoglycan proteins, respectively (1). Recently LGMD2D, LGMD2E, LGMD2F, and LGMD2C are reclassified as limb-girdle muscular dystrophy recessive type 3 (LGMDR3), LGMDR4, LGMDR6, and LGMDR5, respectively (2). A tetrameric subcomplex of four sarcoglycans forms part of the dystrophin-glycoprotein complex (DGC) across the sarcolemma of skeletal and cardiac muscle fibers, which maintains muscle membrane stability during cycles of muscle contraction and relaxation (3). Pathogenic variants in genes encoding DGC components disrupt stability and integrity of the whole complex, leading to the so-called DGC-related muscular dystrophies which comprise sarcoglycanopathies, dystrophinopathies, and α-dystroglycanopathies (4).

Sarcoglycanopathies cover a broad clinical spectrum, ranging from a mild phenotype of asymptomatic increase in serum concentration of creatine kinase (CK) to a severe Duchenne-like muscular dystrophy phenotype, which is overlapping with dystrophinopathies and α-dystroglycanopathies (5). Furthermore, both reduced expression of sarcoglycans and dystrophin are observed in sarcoglycanopathies and dystrophinopathies (5, 6); this makes it difficult to accurately predict the primary genetic defect among them based on muscle immunoanalysis. Thus, the confirmatory diagnosis of a sarcoglycanopathy or dystrophinopathy mainly relies on genetic testing. However, the precise genetic diagnosis of a sarcoglycanopathy or dystrophinopathy is sometimes extremely challenging, as non-canonical splicing site variants, deep intronic splice-altering variants (DISVs), and/or complex structural variants do exist in *DMD* (7, 8) or sarcoglycan (1, 6, 9–13) genes. To our knowledge, only six non-canonical splicing site variants in *SGCA* (1, 9, 10), *SGCB* (6), and *SGCG* (11, 12) and two DISVs in *SGCA* (1, 13) have been previously reported, of which the identification required mRNA studies and bioinformatic splicing analysis.

In this study, three patients from two unrelated families with a highly suspected sarcoglycanopathy or dystrophinopathy based on their clinical, muscle MRI, and pathological features were enrolled, for whom multiplex ligation-dependent probe amplification (MLPA) and an exomic next-generation sequencing (NGS) panel failed to yield a definite genetic diagnosis. To identify potential non-coding splice-altering variants that might be missed by the panel and MLPA-analysis, muscle-derived mRNA studies of *DMD* and sarcoglycan genes were performed in them. Finally, two novel DISVs and a rare exonic splice-altering variant, i.e., an aberrant splicing event induced by a predicted nonsense variant, were first identified in *SGCB*. Furthermore, we also identified a novel non-canonical splicing variant in *SGCB*. The three patients harboring the pathogenic compound heterozygous variants in *SGCB* were eventually diagnosed with beta-sarcoglycanopathy.

## Materials and Methods

### Patients

**FIGURE 1:**
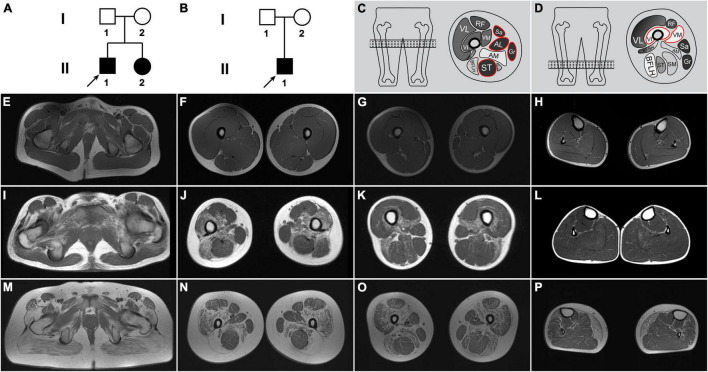
Pedigrees and muscle T1-weighted images of patients with beta-sarcoglycanopathy. **(A)** Family 1. **(B)** Family 2. Each family member was identified with a pedigree number. Affected members colored in black. The black arrow indicated the proband for each family. Schematics of the trefoil with single fruit sign **(C)** and the concentric fatty infiltration pattern **(D)**. **(E–H)** A healthy control. **(I–L)** Patient F1-II1. **(M–P)** Patient F2-II1. **(I)** An axial T1-weighted image of pelvis muscles of patient F1-II1 showing moderate fatty infiltration of the pectineus and obturator externus muscles. Axial T1-weighted images of patient F1-II1 showing moderate fatty infiltration of the adductor longus muscle at proximal thigh level **(J)** and the concentric fatty infiltration pattern around distal femoral diaphysis **(K)**, which consisted of severe fatty infiltration of the vastus medialis and vastus intermedius muscles with sparing of the vastus lateralis muscle **(D)**. **(L)** No obvious muscle fatty infiltration was observed in the lower leg muscles of patient F1-II1. **(M)** Mild fatty infiltration of the pectineus and tensor fasciae latae muscles and severe fatty infiltration of the gluteus maximus muscle were observed in patient F2-II1. Axial T1-weighted images of patient F2-II1 showing the concentric fatty infiltration pattern **(O)** and the trefoil with single fruit sign at proximal thigh level **(N)**, which consisted of three leaflets formed by relative sparing of the adductor longus, sartorius, and gracilis muscles and one single fruit formed by sparing of the semitendinosus muscle **(C)**. **(P)** Severe fatty infiltration of the gastrocnemius medialis muscle was observed in patient F2-II1. RF, rectus femoris; VL, vastus lateralis; VI, vastus intermedius; VM, vastus medialis; Sa, sartorius; AL, adductor longus; Gr, gracilis; AM, adductor magnus; BFSH, biceps femoris, short head; BFLH, biceps femoris, long head; ST, semitendinosus; SM, semimembranosus.

Three patients who had an elevated serum CK level and/or progressive muscle weakness were enrolled in this study, including patients F1-II1 and F1-II2 from family 1 (F1-II1 and F1-II2 are siblings) and patient F2-II1 from family 2 (Figures 1A,B). Clinical features were ascertained by review of the medical records and a detailed physical examination. Walking ability was recorded as follows: hyperCKemia with or without exercise-induced myalgia, running with difficulties, unable to run, ambulant with support, and non-ambulant. Each muscle group strength was graded by manual muscle testing. Total muscle strength was calculated using a conversion formula of Medical Research Council (14).

The electrocardiogram and echocardiography examinations were performed in patients F1-II1 and F2-II1. Cardiac MRI, including late gadolinium enhancement imaging for scar evaluation, was performed in patient F2-II1. MRI examination of the pelvis, thigh, and lower leg muscles was performed in a healthy control and patients F1-II1 and F2-II1 according to a previously described protocol (5). The extent of fatty infiltration of each individual muscle was assessed and scored on axial T1-weighted images using a modified Mercuri’s scale with 0–5 scores (5). Cumulative scores for fatty infiltration of the pelvis, thigh, and lower leg muscles were calculated in patients F1-II1 and F2-II1.

### Muscle Biopsy

Muscle biopsies were obtained from tibialis anterior in patient F2-II1 and biceps brachii in patient F1-II1 and a healthy control subject. The biopsy samples were frozen in isopentane, then cooled in liquid nitrogen, and finally stored at −80°C. Routine histological, histochemical, and immunohistochemical staining were performed in them according to standard protocols (15). Primary monoclonal antibodies against the dystrophin and sarcoglycan proteins were used, including dystrophin-N (amino-terminal), dystrophin-R (rod-domain), dystrophin-C (carboxyl-terminal), α-sarcoglycan, β-sarcoglycan, and γ-sarcoglycan (Novocastra Laboratories, Newcastle) (5, 15).

### Routine Genomic Detection Approaches

Multiplex ligation-dependent probe amplification-analysis of *DMD*, *SGCA*, *SGCB*, *SGCD*, and *SGCG* (8) was performed in the three patients to detect large exonic deletions or duplications. To detect single-nucleotide variants (SNVs) and small insertions/deletions, we performed an exomic NGS panel (15) in them, which covers exons and flanking intronic sequences of genes known to be associated with Mendelian neuromuscular disorders. Sanger sequencing was performed to validate the genomic variants detected by the panel.

### Muscle-Derived mRNA Studies and Genomic Sanger Sequencing

Total muscle RNA was extracted from the remaining muscle tissue using an RNA extraction kit (Invitrogen, La Jolla, CA, United States) and subsequently retrotranscribed to cDNA using a HiScript II Q RT SuperMix kit (Vazyme, Nanjing, China) according to the previous protocols (8, 16). Full length cDNA of *DMD* (NM_004006.2), *SGCA* (NM_000023.2), *SGCB* (NM_000232.4), *SGCD* (NM_000337.5), and *SGCG* (NM_000231.2) were, respectively, amplified and Sanger sequenced in overlapping cDNA fragments using a set of primers (Supplementary Tables 1, 2). Aberrant cDNA fragments were analyzed by gel electrophoresis, and normal and blank controls were included in each analysis.

TA cloning was performed when the direct reverse transcription-polymerase chain reaction (RT-PCR) analysis of cDNA fragments failed to identify various transcripts. The purified PCR products of the aberrant transcripts were reacted with a T vector assay (pClone007 Cloning Vector Kit, TSV-007, Tsingke, Beijing, China) and then cloned into competent cells. After the reaction on ice, in a water bath with 42°C, and on ice again, the reaction was incubated in Lysogeny Broth medium. The competent cells were spread on the plate and grown overnight at 37°C. Selected colonies were inoculated in Lysogeny Broth/Ampicillin medium and shaken overnight at room temperature. The plasmids were digested with restriction enzymes and then Sanger sequenced using the primers M13R (CAGGAAACAGCTATGACC) and M13F (TGTAAAACGACGGCCAGT) (17). Genomic Sanger sequencing around the sites from which aberrant transcripts were produced was performed to detect possible splice-altering variants. The primers used for the amplification and sequencing of the genomic DNA were described in Supplementary Table 3. Segregation studies, including the detected genomic variants and phenotypes, were performed in both families.

### Bioinformatic Analyses

The Human BLAT Search tool was used to search genomic sequences (GRCh37/hg19) that were homologous to the aberrant transcripts. The Human Splicing Finder tool (18) was used to predict alterations in essential splicing signals and splicing regulatory elements caused by the detected genomic variants. The genomic variants, RNA variants, and protein variants identified in this study were described according to the Human Genome Variation Society nomenclature (19). Pathogenicity of each detected genomic variant was interpreted and classified according to the American College of Medical Genetics (ACMG) guidelines (20).

## Results

### Clinical, Muscle MRI, and Pathological Characteristics

**FIGURE 2:**
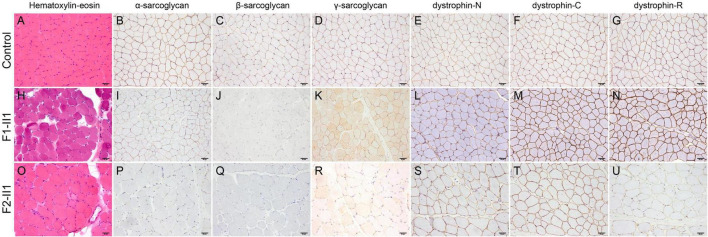
Pathologic features of patients with beta-sarcoglycanopathy. **(H,O)** Hematoxylin-eosin staining showing a muscular dystrophic pattern in patients F1-II1 and F2-II1. **(A–G)** A normal control showing positive expression of sarcoglycans and dystrophin. **(H–N)** Patient F1-II1 showing a mild reduction of α-sarcoglycan, complete deficiency of β-sarcoglycan, a partial reduction of γ-sarcoglycan, a very slight reduction of dystrophin-N, and positive expression of dystrophin-C and dystrophin-R. **(O–U)** Patient F2-II1 showing a severe reduction of α-sarcoglycan, absent expression of β-sarcoglycan, a partial reduction of γ-sarcoglycan, a very slight reduction of dystrophin-N, positive expression of dystrophin-C, and a partial reduction of dystrophin-R. 200× magnification.

Patient F1-II1 is a 14-year-old adolescent boy who presented progressive lower limb weakness since 5-years of age. He had a positive Gowers’ sign at 7-years of age and waddling gait at 12-years of age. Physical examination confirmed that he had proximal muscle weakness with total muscle strength of 91%, calf hypertrophy, and moderate bilateral tendon contractures. Currently, at 14-years of age, he has difficulties in running and no cardiac involvement in terms of clinical manifestations, electrocardiogram, and echocardiography. His serum CK level was markedly elevated in every test (range 7,259–10,085 IU/L; normal 25–170 IU/L). His muscle MRI examination showed moderate muscle fatty infiltration of the pelvis and thigh muscles compared to a healthy control (Figures 1E–J), and no obvious muscle fatty infiltration of the lower leg muscles (Figure 1L). Moreover, his muscle MRI examination showed a distinctive muscle involvement pattern, the concentric fatty infiltration pattern around distal femoral diaphysis (Figures 1D,K), which is highly specific for a sarcoglycanopathy (5). The cumulative score for fatty infiltration of the lower extremity muscles was 37. His muscle biopsy and immunohistochemical staining revealed a muscular dystrophic pattern, a mild reduction of α-sarcoglycan, complete deficiency of β-sarcoglycan, a partial reduction of γ-sarcoglycan, a very slight reduction of dystrophin-N, and positive expression of dystrophin-C and dystrophin-R compared to a normal control (Figures 2A–N).

Patient F1-II2 is a 4-year-old girl who presented to our hospital because of her brother’s admission. She has an elevated level of serum CK (4,210 IU/L). She has no muscle weakness, calf hypertrophy, or tendon contractures confirmed by the physical examination. She did not undergo a muscle MRI examination or muscle biopsy, as it was performed in her brother, patient F1-II1.

Patient F2-II1 is a 17-year-old adolescent boy. He presented to our hospital at 17-years of age because of exercise-induced myalgia and myalgia since 9-years of age. He was confirmed to have a sensorineural hearing loss at 10-years of age. He had a positive Gowers’ sign at 11-years of age and waddling gait at 14-years of age. He had suffered from chest distress and exercise intolerance since 14-year-old. Physical examination confirmed that he had both proximal and distal muscle weakness with total muscle strength of 77%, calf hypertrophy, and severe bilateral tendon contractures. He is now unable to run or complete the Gowers’ maneuver and confirmed to have a dilated cardiomyopathy. His electrocardiogram showed a right bundle branch block. His echocardiography revealed left ventricular dilatation, diffuse hypokinesis of left ventricular wall motion, and reduced cardiac output with a left ventricular ejection fraction of 41%. His cardiac muscle MRI confirmed wall motion abnormalities, left ventricular enlargement, and partial fibrosis with an epicardial and mesocardial scar along the inferior and lateral walls of the left ventricle. His serum CK level was elevated in every test, ranging from 1,750 to 10,000 IU/L. His muscle MRI examination revealed mild fatty infiltration of the pectineus and tensor fasciae latae muscles and severe fatty infiltration of the gluteus maximus muscle (Figure 1M). Furthermore, his muscle MRI examination showed not only the concentric fatty infiltration pattern (Figure 1O) but also the trefoil with single fruit sign at proximal thigh level that is highly specific for a dystrophinopathy (Figures 1C,N). Severe fatty infiltration of the gastrocnemius medialis muscle was observed in patient F2-II1 (Figure 1P). The cumulative score for fatty infiltration was 58. His muscle biopsy and immunohistochemical staining showed a muscular dystrophic pattern, a severe reduction of α-sarcoglycan, absent expression of β-sarcoglycan, a partial reduction of γ-sarcoglycan, a very slight reduction of dystrophin-N, positive expression of dystrophin-C, and a partial reduction of dystrophin-R (Figures 2O–U).

Based on the clinical phenotypes, family history, and muscle MRI and pathological features, the three patients enrolled in this study were highly suspected of having a sarcoglycanopathy or dystrophinopathy.

### Pathogenic Variants Identified in This Study

#### Variants Detected in Routine Genomic Detection Approaches

Multiplex ligation-dependent probe amplification-analysis of *DMD*, *SGCA*, *SGCB*, *SGCD*, and *SGCG* didn’t detect any large exonic deletions or duplications in the three patients. The NGS panel detected a variant of uncertain significance in the non-canonical splicing site region of *SGCB*, the c.243 + 6T > A variant in patients F1-II1 and F1-II2 (Table 1). In patient F2-II1, the NGS panel detected a heterozygous nonsense variant in *SGCB*, the c.334C > T, p.(Gln112*) variant, and a homozygous frameshift variant in *GJB2*, NM_004004.5:c.235del, p.(Leu79Cysfs*3). The *GJB2* variant has been previously reported as a pathogenic variant responsible for autosomal recessive non-syndromic hearing impairment (21). In addition, c.334C > T has also been reported as a pathogenic *SGCB* variant (15).

**TABLE 1:** Pathogenic *SGCB* variants identified in our patients with beta-sarcoglycanopathy.

Patient number	Intron/Exon	Genomic variant	Parental derivation	cDNA variant	RNA variant	Protein variant	Evidence of pathogenicity
F1-II1	Intron 2	g.52898039G > A	Paternal	c.243 + 1558C > T	r.243_244ins243 + 1470_243 + 1556	p.Ile82Leufs*24	PVS1, PM2, PP1, PP4
	Intron 2	g.52899591A > T	Maternal	c.243 + 6T > A	r.243_244ins[243 + 1_243 + 10;243 + 6u > a]	p.Ile82Valfs*20	PVS1, PM2, PP1, PP4
F1-II2	Intron 2	g.52898039G > A	Paternal	c.243 + 1558C > T	NA	NA	Same as his brother F1-II1
	Intron 2	g.52899591A > T	Maternal	c.243 + 6T > A	NA	NA	Same as his brother F1-II1
F2-II1	Exon 3	g.52895939G > A	Paternal	c.334C > T	r.333_429del	p.Gln112Leufs*12	PVS1, PM2, PP4
	Intron 2	g.52898021G > C	Maternal	c.243 + 1576C > G	r.243_244ins243 + 1470_243 + 1575	p.Ile82Leufs*24	PVS1, PM2, PP4

Pathogenic SGCB variants were described in relation to the SGCB gene genomic reference sequence (NC_000004.11, genome build GRCh37), coding DNA reference sequence (NM_000232.4), RNA reference sequence (NM_000232.4), and protein reference sequence (NP_000223.1). NA, muscle biopsy and muscle-derived RNA studies were not performed in the patient. ins, insertion; del, deletion; *, stop codon. PVS1, all the four SGCB variants were frameshift variants confirmed by our mRNA studies (very strong evidence); PM, moderate evidence; PP, supporting evidence.

#### Variants Identified *via* mRNA Studies and Genomic Sanger Sequencing

**FIGURE 3:**
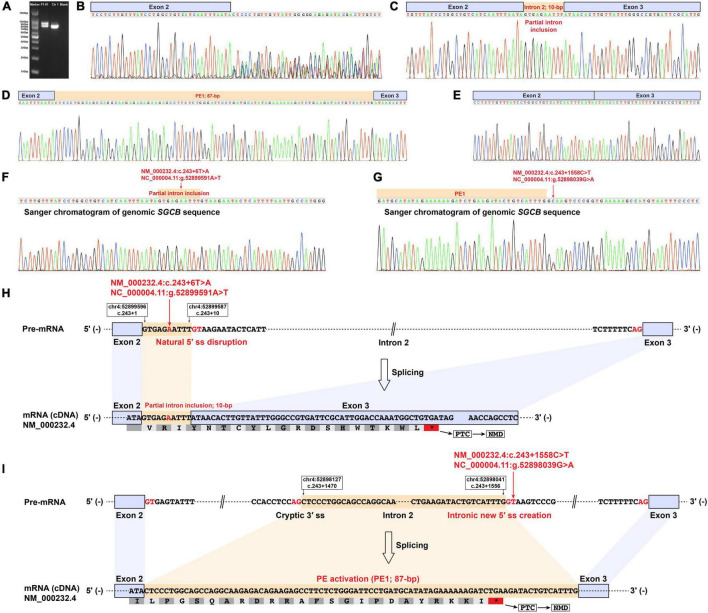
Muscle-derived mRNA studies and genomic *SGCB* sequencing of patient F1-II1. **(A)** RT-PCR amplification of the aberrant *SGCB* transcripts from patient F1-II1 showed that the upper band was larger than the expected band, while the lower band was almost the same size as the expected band. **(B)** Direct Sanger sequencing of the aberrant *SGCB* transcripts could not recognize the overlapping sequences. **(C–E)** TA cloning of the aberrant cDNA fragments revealed a 10-bp sequence insertion and an 87-bp insertion of intron 2 sequence between *SGCB* exons 2 and 3 (PE1), and the normal splicing of *SGCB* exons 2–3. **(F)** Sanger sequencing of the genomic sequence around *SGCB* exon 2 revealed the c.243 + 6T > A variant. **(G)** Sanger sequencing of the genomic *SGCB* sequence around the PE1 revealed the c.243 + 1558C > T variant. **(H)** The schematic of the aberrant insertion of 10-bp sequence between *SGCB* exons 2 and 3 caused by the c.243 + 6T > A variant in *SGCB*. **(I)** The schematic of the aberrant 87-bp insertion of intron 2 sequence between *SGCB* exons 2 and 3 caused by the c.243 + 1558C > T variant in *SGCB*. Ctr1, a normal control; Blank, a reagent control; PE, pseudoexon; RT-PCR, reverse transcription-polymerase chain reaction; F, fragment; bp, base pair; PTC, premature termination codon; NMD, nonsense-mediated decay.

The RT-PCR analysis of *DMD*, *SGCA*, *SGCD*, and *SGCG* mRNA didn’t reveal any aberrant transcripts in patients F1-II1 and F2-II1. Alterations in splice site strength caused by splice-altering variants in *SGCB* were summarized in Supplementary Table 4. RT-PCR amplification of the *SGCB* exons 1–5 from patient F1-II1 showed that the upper band was larger than the expected band, while the lower band was almost the same size as the expected band (Figure 3A). Direct Sanger sequencing of the aberrant *SGCB* transcripts could not recognize the overlapping sequences (Figure 3B). Thus, TA cloning of the aberrant cDNA fragments was performed, which revealed two aberrant *SGCB* splicing events, i.e., a 10-base pair (bp) sequence insertion (Figure 3C) and an 87-bp insertion of intron 2 sequence (pseudoexon 1, PE1) between *SGCB* exons 2 and 3 (Figure 3D), and the normal splicing of *SGCB* exons 2–3 (Figure 3E). Sanger sequencing of the genomic SGCB sequence around exon 2 and the PE1 revealed the c.243 + 6T > A variant (Figure 3F) and a deep intronic SNV, c.243 + 1558C > T, adjacent to the PE1 (Figure 3G). The non-canonical splicing site variant, c.243 + 6T > A, disrupted the natural donor splice site (5′ ss) of exon 2 and activated a cryptic 5′ ss in intron 2, resulting in the partial intron inclusion of a 10-bp sequence into the mature *SGCB* transcript (Figure 3H and Supplementary Table 4). The c.243 + 1558C > T variant created a new 5′ ss in intron 2 that was paired with the adjacent cryptic acceptor splice site (3′ ss), causing the activation of PE1 (Figure 3I and Supplementary Table 4). Both the partial intron inclusion and PE1 activation transcripts encoded a frameshift and premature termination codon (Table 1) that were targeted for degradation by nonsense-mediated decay (NMD), leading to the complete deficiency of β-sarcoglycan observed in patient F1-II1 (Figure 2J).

**FIGURE 4:**
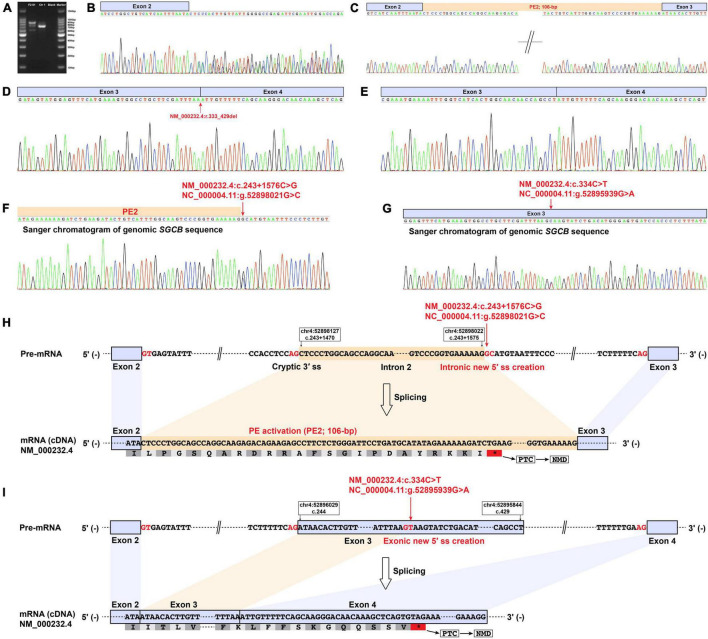
Muscle-derived mRNA studies and genomic *SGCB* sequencing of patient F2-II1. **(A)** RT-PCR amplification of the aberrant *SGCB* transcripts from patient F2-II1 showed three bands with varying size. **(B)** Direct Sanger sequencing of the aberrant *SGCB* transcripts could not recognize the overlapping sequences. **(C–E)** TA cloning of the aberrant cDNA fragments revealed a 106-bp insertion of intron 2 sequence between *SGCB* exons 2 and 3 (PE2), a 97-bp truncation of *SGCB* exon 3, and the normal splicing of *SGCB* exons 3–4. **(D)** The Sanger chromatogram showed the beginning sequences of *SGCB* exon 3 and indicated the 97-bp truncation of *SGCB* exon 3 (NM_000232.4:r.333_429del). **(F)** Sanger sequencing of the genomic *SGCB* sequence around the PE2 revealed the c.243 + 1576C > G variant. **(G)** Sanger sequencing of the genomic *SGCB* exon 3 sequence revealed the c.334C > T variant. **(H)** The schematic of the aberrant 106-bp insertion of intron 2 sequence between *SGCB* exons 2 and 3 caused by the c.243 + 1576C > G variant in *SGCB*. **(I)** The schematic of the aberrant 97-bp truncation of *SGCB* exon 3 caused by the c.334C > T variant in *SGCB*. Ctr1, a normal control; Blank, a reagent control; PE, pseudoexon; RT-PCR, reverse transcription-polymerase chain reaction; F, fragment; bp, base pair; PTC, premature termination codon; NMD, nonsense-mediated decay.

Reverse transcription-polymerase chain reaction amplification of the *SGCB* exons 1–5 from patient F2-II1 showed three bands with varying size (Figure 4A). Direct Sanger sequencing of the aberrant *SGCB* transcripts could not recognize the overlapping sequences (Figure 4B). TA cloning of the aberrant cDNA fragments was therefore performed and revealed two aberrant *SGCB* splicing events, i.e., a 106-bp insertion of intron 2 sequence between *SGCB* exons 2 and 3 (PE2; Figure 4C) and a 97-bp truncation of *SGCB* exon 3 (Figure 4D), and the normal splicing of *SGCB* exons 3–4 (Figure 4E). Sanger sequencing of the genomic *SGCB* sequence around the PE2 and exon 3 revealed a deep intronic SNV, c.243 + 1576C > G, adjacent to the PE2 (Figure 4F), and the c.334C > T variant (Figure 4G). The c.243 + 1576C > G variant created a new 5′ ss in intron 2 that was paired with the adjacent cryptic 3′ ss, resulting in the activation of PE2 (Figure 4H and Supplementary Table 4). Unexpectedly, the predicted nonsense variant, c.334C > T, p.(Gln112*) in *SGCB*, created a new 5′ ss in exon 3 that was stronger than the natural 5′ ss of exon 3, eventually causing a 97-bp deletion of *SGCB* exon 3 (Figure 4I and Supplementary Table 4). Hence, the predicted nonsense variant is actually an exonic splice-altering variant. Both the PE2 activation and exon truncation transcripts encoded a frameshift and premature termination codon (Table 1), which were targeted for degradation by NMD and resulted in the absent expression of β-sarcoglycan observed in patient F2-II1 (Figure 2Q).

The two DISVs (c.243 + 1558C > T and c.243 + 1576C > G) and one non-canonical splicing site variant (c.243 + 6T > A) in *SGCB* identified in this study are novel variants, as they are not reported in the literature and absent from population and disease-specific databases (20), including the Genome Aggregation Database, ClinVar, and Leiden Open Variation Database. Segregation studies confirmed that c.243 + 1558C > T and c.243 + 6T > A are compound heterozygous variants in patients F1-II1 and F1-II2, and c.243 + 1576C > G and c.334C > T in patient F2-II1. All four *SGCB* variants identified in our patients are classified as a pathogenic variant according to the ACMG guidelines (20), as each of them fulfills the criteria described in Table 1. The three patients harboring the pathogenic compound heterozygous variants in *SGCB* were eventually diagnosed with beta-sarcoglycanopathy.

## Discussion

In this study, three patients from two unrelated families were highly suspected of a sarcoglycanopathy or dystrophinopathy based on their clinical, muscle MRI, and pathological features, which drove us to perform muscle-derived mRNA studies of *DMD* and sarcoglycan genes after indefinite findings in routine genetic testing in them. Finally, we identified rare intronic and exonic splice-altering variants in *SGCB* and confirmed the genetic diagnosis of them. These cases are an example of how clinical, radiological, and pathological data can facilitate the further genetic testing in rare Mendelian diseases.

The confirmatory diagnosis of a sarcoglycanopathy or dystrophinopathy relies primarily on genetic testing, as they share overlapping characteristics in clinical, radiological, and pathological features (5). To date, there is no proven curable treatment for sarcoglycanopathy or dystrophinopathy. Therefore, prenatal diagnosis that requires precise detection of pathogenic variants in *DMD* or sarcoglycan genes can be of great value for families with a sarcoglycanopathy or dystrophinopathy. Like the other monogenic diseases, most of the pathogenic variants in *DMD* or sarcoglycan genes are in coding regions or canonical splice sites (4, 8, 12, 15, 22), which can be detected by routine genomic detection approaches. However, pathogenic non-coding variants including non-canonical splicing site variants and DISVs do exist in *DMD* (7, 8) or sarcoglycan (1, 6, 9–13) genes, which are simply missed by the routine genomic detection approaches. The identification of non-coding splicing variants requires mRNA studies and bioinformatic splicing analysis in addition to the genomic sequencing (8, 23). Therefore, after indefinite findings in routine approaches in three patients with a suspected sarcoglycanopathy or dystrophinopathy, we performed muscle-derived mRNA studies of *DMD* and sarcoglycan genes and relevant bioinformatic splicing analysis in them, including direct RT-PCR analysis and TA cloning. As the direct RT-PCR analysis of *SGCB* mRNA failed to identify the various transcripts observed, TA cloning was used in this study. Finally, we successfully identified three novel intronic and one exonic splice-altering variants in *SGCB via* the combination of mRNA studies and genomic Sanger sequencing, which confirmed the genetic diagnosis of them. The novel intronic *SGCB* variants identified in our patients emphasizes the potential role of underdetected DISVs in rare Mendelian diseases.

To our knowledge, only six non-canonical splicing site variants, including four in *SGCA* (1, 9, 10), one in *SGCB* (6), and two in *SGCG* (11, 12), and two DISVs in *SGCA* (1, 13) have been previously reported. Similar to the previously reported non-canonical splicing site variant in intron 2 of *SGCB* (c.243 + 5G > A) (6), the c.243 + 6T > A identified in our study is also located in intron 2. Different to the two reported DISVs in *SGCA*, i.e., the c.585-31_585-24del and c.37 + 23G > A variants that are not too far from the exon-intron junctions (1, 13), the two novel DISVs in *SGCB* (c.243 + 1558C > T and c.243 + 1576C > G) identified in our study are quite far from the exon-intron junctions, which is the first identification of DISVs in *SGCB*. All the reported and newly discovered non-canonical splicing site variants and DISVs in *SGCB* are located in intron 2, suggesting that intron 2 is a hotspot region for intronic variants in β-sarcoglycanopathy. Genetic investigation of the region (chr4:52898000-52899000; GRCh37/hg19) should be performed in genetically undiagnosed patients with a highly suspected sarcoglycanopathy. Unexpectedly, we found that a reported nonsense variant in *SGCB*, the c.334C > T variant, is actually an exonic splice-altering variant, as it created a new donor splice site in exon 3 that was stronger than the natural one and resulted in the exon truncation followed by NMD, which is also the first identification of exonic splice-altering variant in *SGCB*. All the aberrant *SGCB* transcripts identified in our patients encoded a frameshift and premature termination codon followed by NMD and resulted in the complete deficiency of β-sarcoglycan. This indicates that in addition to coding variants that can directly change a protein sequence, aberrant pre-mRNA splicing that induced by intronic or exonic splice-altering variants can also be devastating for the encoded protein.

The clinical characteristics of our patients with β-sarcoglycanopathy are similar to the findings of previous studies (1, 10, 15, 24, 25). We find that patients with β-sarcoglycanopathy may also have another Mendelian disease, like the sensorineural hearing loss phenotype caused by pathogenic *GJB2* variants observed in patient F2-II1. Presence of the two distinct Mendelian forms, i.e., β-sarcoglycanopathy and *GJB2*-related hearing impairment, in a same patient highlights the significance of genetic counseling in patients with β-sarcoglycanopathy. Clinical geneticists, pediatricians, and neurologists should be aware of this possibility. Our previous studies have confirmed that the concentric fatty infiltration pattern around distal femoral diaphysis, a selective fatty infiltration pattern observed on muscle MRI, is highly specific for sarcoglycanopathies with pathogenic coding variants in sarcoglycan genes (5). This study first confirms that β-sarcoglycanopathy with pathogenic non-coding variants can also present the concentric fatty infiltration pattern. In addition, patient F2-II1 with β-sarcoglycanopathy showed both the concentric fatty infiltration pattern and the trefoil with single fruit sign that is highly specific for a dystrophinopathy. These overlapping muscle MRI patterns indicate that different defects in the components of DGC can cause impairment of the sarcolemma integrity and stability (3), which might lead to some similarities among the affected skeletal muscles (5).

In conclusion, we successfully identified three novel intronic and one exonic splice-altering variants in *SGCB via* the combination of muscle-derived mRNA studies and genomic Sanger sequencing, which is the first identification of rare exonic and DISVs in *SGCB*. Our study expands the clinical and genetic spectrum of β-sarcoglycanopathy and indicates that intronic and exonic splice-altering variants are also important causes of sarcoglycanopathies.

## Data Availability Statement

The datasets presented in this study can be found in online repositories. The names of the repository/repositories and accession number(s) can be found in the article/Supplementary Material.

## Ethics Statement

The studies involving human participants were reviewed and approved by the Ethics Committee at Peking University First Hospital. Written informed consent to participate in this study was provided by the participants’ legal guardian/next of kin. Written informed consent was obtained from the minor(s)’ legal guardian/next of kin for the publication of any potentially identifiable images or data included in this article.

## Author Contributions

ZX, CS, WZ, and YY conceived and planned the study, take full responsibility for the manuscript, and contributed to the revisions of the manuscript. ZX, CS, and WZ contributed to the methodology of the study. ZX, CS, MY, YZ, LM, FL, and YL conducted the clinical and radiological study. ZX, CS, CL, XC, QG, and JD conducted the genetic research. DX and LW performed the experiments of muscle-derived RNA studies. ZX and ZW checked all the genetic analyses. ZX and CS took the lead in writing the manuscript. WZ and YY supervised the study. All authors contributed to the article and approved the submitted version.

## Conflict of Interest

DX and LW were employed by Running Gene Inc., Beijing, China. They performed the experiments of muscle-derived RNA studies. The remaining authors declare that the research was conducted in the absence of any commercial or financial relationships that could be construed as a potential conflict of interest.

## Publisher’s Note

All claims expressed in this article are solely those of the authors and do not necessarily represent those of their affiliated organizations, or those of the publisher, the editors and the reviewers. Any product that may be evaluated in this article, or claim that may be made by its manufacturer, is not guaranteed or endorsed by the publisher.
