# Supplementary material for: First Identification of Rare Exonic and Deep Intronic Splice-Altering Variants in Patients With Beta-Sarcoglycanopathy
Source: Front Pediatr. 2022 Jun 22;10:900280. doi: 10.3389/fped.2022.900280 (PMC9257024; doi:10.3389/fped.2022.900280)
Supplement: Supplementary file 1 [file Data_Sheet_1.PDF]

Supplementary Table 1. The list of primers used for the RT-PCR amplification of *DMD* mRNA.

Amplified fragments	Size (bp)	Forward primer sequence (5′ -> 3′)	Reverse primer sequence (5′ -> 3′)
F1 (exons 1~ 7)	606	ACTGGAGCAATAAAGTTTGAAGAAC	CTGGCCTATGACTATGGATGAGA
F2 (exons 6~10)	597	AAGATTCTCCTGAGCTGGGTC	CTCCATCAATGAACTGCCAAA
F3 (exons 9~13)	649	GGCTGCTTATGTCACCACCTCT	TTCATCAACTACCACCACCATG
F4 (exons 12~17)	686	AGTACAACAACATAAGGTGCTTCA	AATCCACAGTAATCTGCCTCTTC
F5 (exons 17~21)	631	CACCACTCAGCCATCACTAACA	TAGCCGGTTGACTTCATCCTTA
F6 (exons 20~24)	725	CAGAACAACATCATCGCTTTCTAT	AAAACATCAACTTCAGCCATCC
F7 (exons 23~27)	618	AAATTGAGGGACGCTGGAA	GTGGAGCTTGAGCTATGACACTA
F8 (exons 26~30)	701	CTGTAAGCCTCCAGAAAGAT	CTGCTTGTCAATGAATGTGA
F9 (exons 30~34)	696	GAATCCAGGAGTCCCTCACAT	CAGGCAACTTCAGAATCCAAA
F10 (exons 34~37)	567	ATTGTCCCGTAAGATGCGAAAG	AGCTCTGAGATTTGGGGCTCTA
F11 (exons 37~41)	625	ATACGCCCAAAGGTGGACTC	CCTCAGCTTGCCTACGCACT
F12 (exons 40~44)	606	CAAATTAGCCAGCCTACCTG	GTCAAATCGCCCTTGTCG
F13 (exons 43~47)	671	TATTCATAGCAAGAAGACAGCAGCAT	GCACGGGTCCTCCAGTTTCA
F14 (exons 46~50)	592	GATAACATTGCTAGTATCCCACTT	CTAGGTCAGGCTGCTTTGC
F15 (exons 49~53)	527	AAAACCAGCCACTCAGCC	TGGTGTTCTTGTACTTCATCCC
F16 (exons 52~55)	571	AAAACAAGACCAGCAATCAAG	GAGTCTTCTAGGAGCCTTTCC
F17 (exons 55~59)	632	AAGTTTCTTGCCTGGCTTACA	TCCTCAGCCTGCTTTCGTAG
F18 (exons 58~63)	623	GACAGAGCAGCCTTTGGAAG	TTGTTTGAGTCTCGTGGTTGATA
F19 (exons 61~67)	627	GGACTTTGGTCCAGCATCTCA	GCAACTTCACCCAACTGTCTTG

F20 (exons 65~70)	633	CGGGACGAACAGGGAGGAT	TCTGCACTGGCAGGTAGCC
F21 (exons 69~75)	628	TTGCACTCCGACTACATCAGG	GTGTTGACGCAGTAGCTTGG
F22 (exons 75~79)	509	CTAAAGCAGCAGCACGAACA	CAAATCATCTGCCATGTGGA

Supplementary Table 2. The list of primers used for the RT-PCR amplification of *SGCA*, *SGCB*, *SGCD*, and *SGCG* mRNA.

Gene	Amplified fragments	Forward primer sequence (5′ -> 3′)	Reverse primer sequence (5′ -> 3′)
SGCA	F1 (exons 1~ 6)	TGGCTGAGACACTCTTCTGG	TCGTAGCAAGACAGAAGTGGAG
SGCA	F2 (exons 5~10)	CTTCCCATTGAGGGCCGAAA	CTCGTGGTGTGGACAGAGAA
SGCB	F3 (exons 1~5)	GGCTGCAGAACAGCAAAG	TCATTTCCACGCACAATAGC
SGCB	F4 (exons 4~6)	AGTTTCATTTGCCAAGTGGAGT	CAGCTGCTTCAGACCTTTAACC
SGCD	F5 (exons 1~8)	AGTGGCCACCTCCTTCAGAG	ATCACTAGAGACCGGGTTGG
SGCD	F6 (exons 6~10)	CTCTGCAGACAATAATGAAGTGG	AGTGGATATCGCAGGGTCAC
SGCG	F7 (exons 1~6)	AAGCTTCATCCTTTGCTCTCA	AGGGGTGTCTCCACTGAATG
SGCG	F8 (exons 4~8)	GAGGTCACAGGCAGGTTAAA	TGCCTCAGTCTTTCACGATG

Supplementary Table 3. The primers used for the amplification and sequencing of the genomic DNA around the sites from which aberrant transcripts were produced.

Gene	Variants	Forward primer sequence (5′ -> 3′)	Reverse primer sequence (5′ -> 3′)
SGCB	c.243+1558C>T	TTCTTGGCACTTTCCTCAAAA	CAGCTACCCCTCCTAATGTCA
SGCB	c.243+6T>A	AAGGGATAAACCCATGCACA	AAATGCACCAAAACGAGAGG
SGCB	c.334C>T	CCATTGAACAAAATCTCA	CCTACCAACAGTGGACTT

SGCB	c.243+1576C>G	TTCTTGGCACTTTCCTCAAAA	CAGCTACCCCTCCTAATGTCA

Supplementary Table 4. Alterations in splice site strength caused by pathogenic splice-altering variants in the *SGCB* gene.

Pathogenic variants	Wild-type 3′ splice site		Mutated 3′ splice site		Wild-type 5′ splice site		Mutated 5′ splice site	
	HSF	MaxEnt	HSF	MaxEnt	HSF	MaxEnt	HSF	MaxEnt
c.243+6T>A	–		–		`ATA|GTGAGT`		`ATA|GTGAG`**`A`**	
	–	–	–	–	81.18	6.97	78.5	1.69
c.243+1558C>T	`TCCTCATTCTCCACCTCCAG|CTC`		–		`TTG|GCAAGT`		`TTG|G`**`T`**`AAGT`	
	84.76	9.12	–	–	65.6	2.72	92.74	10.47
c.243+1576C>G	`TCCTCATTCTCCACCTCCAG|CTC`		–		`AAG|CCATGT`		`AAG|`**`G`**`CATGT`	
	84.76	9.12	–	–	<60	-6.24	65.52	2.03
c.334C>T	–		–		`TAA|GCAAGT`		`TAA|G`**`T`**`AAGT`	
	–	–	–	–	<60	0.31	88.41	8.07

The consensus value and threshold for HSF[1] and MaxEnt[2] were calculated and explained according to the online HSF tool. Margins for the HSF algorithm are between 0 and 100, with a higher score suggesting a greater potential for a donor or acceptor splice site. The MaxEnt algorithm adopts a log odds ratio as the consensus value with different ranges, which depends on the input sequence. The lower the log odds ratio, the more the input sequence could be interpreted as a weaker donor or acceptor splice site. The mutated nucleotides were underlined and in boldface. HSF, human splicing finder; MaxEnt, maximum entropy.

Supplementary References

1. Desmet FO, Hamroun D, Lalande M, Collod-Béroud G, Claustres M, Béroud C. Human Splicing Finder: an online bioinformatics tool to predict splicing signals. Nucleic Acids Res. 2009;37(9):e67.
2. Yeo G, Burge CB. Maximum entropy modeling of short sequence motifs with applications to RNA splicing signals. J Comput Biol. 2004;11(2-3):377-394.
